# Preperations of Iron

**Published:** 1854-11

**Authors:** 


*Preparotions of Iron.*—Donavan's formula is, pure sulphate of iron, one drachm; magnesia, ten grains; purified sugar, one ounce; rose or cinnamon water, eight ounces; mix. This is a scientific prescription; and if the iron be free from oxide, the green color is preserved for eight or ten days. The magnesia neutralizes the sulphuric acid, and converts the sulphate into a protoxide The sugar prevents decomposition, and it may be flavored with mint or peppermint water. In the hysterical female, infusion of vslerian adds to its value; and if there be great sense of exhaustion, ammonia in combination is most beneficial. In occasional constipation, caused by the loss of tone. the sulphate of zinc, with small doses of sulphate of strychnia, relieves. In severe chlorosis, the chrystallized citric acid aids the iron mixture. If there be a periodical neuralgia, the most effective form is the precipitate of corbonate of iron. In severe cases of cholera and anœmic headache, Fowler's solution of arsenic was combined with Donavan's mixture, and in fourteen days both diseases were permanently cured.—*London Lancet.*

